# Host-Selective Toxins of *Pyrenophora tritici-repentis* Induce Common Responses Associated with Host Susceptibility

**DOI:** 10.1371/journal.pone.0040240

**Published:** 2012-07-06

**Authors:** Iovanna Pandelova, Melania Figueroa, Larry J. Wilhelm, Viola A. Manning, Aakash N. Mankaney, Todd C. Mockler, Lynda M. Ciuffetti

**Affiliations:** 1 Department of Botany and Plant Pathology and Center for Genome Research and Biocomputing, Oregon State University, Corvallis, Oregon, United States of America; 2 United States Department of Agriculture, Agricultural Research Service, Forage Seed and Cereal Research Unit, Oregon State University, Corvallis, Oregon, United States of America; 3 The Donald Danforth Plant Science Center, St. Louis, Missouri, United States of America; Soonchunhyang University, Republic of Korea

## Abstract

*Pyrenophora tritici-repentis* (*Ptr*), a necrotrophic fungus and the causal agent of tan spot of wheat, produces one or a combination of host-selective toxins (HSTs) necessary for disease development. The two most studied toxins produced by *Ptr,* Ptr ToxA (ToxA) and Ptr ToxB (ToxB), are proteins that cause necrotic or chlorotic symptoms respectively. Investigation of host responses induced by HSTs provides better insight into the nature of the host susceptibility. Microarray analysis of ToxA has provided evidence that it can elicit responses similar to those associated with defense. In order to evaluate whether there are consistent host responses associated with susceptibility, a similar analysis of ToxB-induced changes in the same sensitive cultivar was conducted. Comparative analysis of ToxA- and ToxB-induced transcriptional changes showed that similar groups of genes encoding WRKY transcription factors, RLKs, PRs, components of the phenylpropanoid and jasmonic acid pathways are activated. ROS accumulation and photosystem dysfunction proved to be common mechanism-of-action for these toxins. Despite similarities in defense responses, transcriptional and biochemical responses as well as symptom development occur more rapidly for ToxA compared to ToxB, which could be explained by differences in perception as well as by differences in activation of a specific process, for example, ethylene biosynthesis in ToxA treatment. Results of this study suggest that perception of HSTs will result in activation of defense responses as part of a susceptible interaction and further supports the hypothesis that necrotrophic fungi exploit defense responses in order to induce cell death.

## Introduction

Plant pathogen interactions that exhibit classical gene-for-gene characteristics have provided a fundamental model for the molecular genetic evaluation of disease development and plant defense. Host resistance (incompatibility) in classical gene-for-gene interactions requires the recognition of the product of a pathogen avirulence (*Avr*) gene by a complementary plant resistance (*R*) gene. Absence of either of these gene products leads to susceptibility (compatibility). The genetic tractability of this model allowed for a better understanding of the role that pathogen effectors’ play in conferring avirulence and their contribution to virulence in the absence of *Avr*/*R* recognition. Continuous efforts have been directed towards determining the mechanisms by which pathogen effectors condition host susceptibility. The identification and characterization of these types of effectors (pathogenicity factors) is integral for understanding the physiological nature of plant disease susceptibility. Fungal pathogens that produce host-selective toxins (HSTs) are ideal organisms to address this issue because HSTs are readily identifiable pathogenicity factors. Cases where a single gene/locus conditions sensitivity to a given HST and susceptibility rather than resistance to the pathogen represent an “inverse” of the classical gene-for-gene interaction [1], [2].

*Pyrenophora tritici-repentis* (*Ptr*) is a necrotrophic fungus and the causal agent of tan spot of wheat, a disease of global economic importance [3]–[8]. *Ptr* isolates may produce one or a combination of HSTs, which result in a pathogen population with a complex race structure [8]–[10]. The resulting susceptibility phenotype depends on the HST(s) produced by the pathogen and the wheat cultivar that supports the pathogen’s growth. HSTs infiltrated into sensitive host genotypes reproduce symptoms triggered by the pathogen [2], [11], [12]. Necrosis, one of the symptoms caused by the most frequently isolated races of *Ptr*, is commonly attributed to the production of Ptr ToxA (ToxA) (syn. Ptr toxin, Ptr necrosis toxin) [10], [13]–[20]; although the presence of other necrosis-inducing activities have been described [18], [21]. ToxA is a 13.2 kDa protein that is encoded by the single copy gene *ToxA* [13]–[15], [17], [18], [20]. A combination of molecular and biochemical evidence demonstrated that ToxA is internalized into toxin sensitive mesophyll cells, and that this process depends on the presence of the RGD motif-containing solvent exposed loop on ToxA and a plant high-affinity receptor [22]–[24]. ToxA-induced cell death is preceded by changes in the photosynthetic machinery, and accumulation of reactive oxygen species (ROS) likely due to the disruption of photosystem (PS) homeostasis [25], [26].

Another proteinaceous HST produced by *Ptr* is Ptr ToxB (ToxB) (syn. Ptr chlorosis toxin), which is responsible for chlorotic symptom development [27]–[29]. ToxB is a 6.5 kDa protein encoded by *ToxB* that must be present in multiple copies to induce strong symptom development [4], [27], [29]–[32]. The mode- and site-of-action for ToxB is not as well-characterized as for ToxA; however, it is known that ToxB-induced chlorosis is light-dependent and involves the disruption of photosynthesis, and possibly the photooxidation of chlorophyll [33]. Similar to ToxA, ROS accumulation has also been hypothesized as part of its mode-of-action; however, experiments with inhibitors of ROS were inconclusive [33], [34]. A recent proteomics analysis to determine the effect of ToxB on a toxin sensitive wheat genotype found that proteins related to ROS detoxification and energy metabolism were amongst the proteins affected by ToxB treatment [34]. ToxB is stable when exposed to organics and heat, and preliminary data indicate that ToxB is resistant to some proteolytic enzymes [28], [30], [35]. Because of these attributes and others shared with apoplastic effectors, including small size and a relatively high cysteine content, we proposed that ToxB can act as an apoplastic effector [30].

As mentioned above, sensitivity to a toxin and susceptibility to the pathogen in the *Ptr*/wheat pathosystem is governed by a single gene/locus. Sensitivity to ToxA is governed by the *Tsn1* gene [36]–[40], and given the strong evidence of a high affinity receptor for ToxA [23], and the requirement for ToxA internalization for activity [22], [23], a reasonable hypothesis was that *Tsn1* encoded the ToxA receptor. However, the recent isolation and characterization of *Tsn1* revealed that Tsn1 lacks a transmembrane domain and does not appear to interact directly with ToxA [38]. Instead, *Tsn1* encodes structural domains commonly associated with two distinct classes of disease resistance (R) genes such as serine/threonine protein kinase (S/TPK) and the nucleotide-binding site–leucine-rich repeat (NBS-LRR) class. The presence of N-terminal S/TPK and C-terminal NBS-LRR domains makes *Tsn1* the first gene characterized with this combination of structural features. Thus, ToxA extends the list of HSTs, like victorin and Pc-toxin [2], [41], [42], whose sensitivity is conferred by host genes related to R-genes, reinforcing the concept that resistance and susceptibility have overlapping signaling pathways. This is, further supported by transcriptome analyses that showed that ToxA treatment of a toxin sensitive wheat genotype elicits reactions similar to those defense responses typically associated with R-gene mediated resistance (i.e. phenylpropanoid and jasmonic acid pathway, ethylene biosynthesis, up-regulation of receptor-like kinases and PR proteins) [43], [44].

Similar to ToxA, sensitivity to ToxB and disease susceptibility are conferred by a single dominant locus in the host [1], [39]. Molecular mapping and QTL analysis identified the locus *Tsc2* and *tsc2* on the short arm of the wheat chromosome 2B, which conditions sensitivity to ToxB and resistance to ToxB producing races, respectively [45]. However, the isolation and characterization of the sensitivity gene, as well as its interaction with ToxB, have not been resolved.

In order to understand the underlying mechanisms of susceptibility, it is critical to determine if common plant responses are triggered by different pathogenicity/virulence factors. The *Ptr*/wheat pathosystem is particularly amenable to these comparative analyses because pathogenic isolates produce multiple toxins with different biochemical characteristics and symptom development. Moreover, these toxins trigger distinct symptoms on the same sensitive wheat cultivar (Katepwa) providing a unique opportunity to study their role in susceptibility by comparing their effects in the same genetic background. In order to determine if ToxA and ToxB induce similar responses and to begin to understand the differences between the mechanisms that induce necrosis vs. chlorosis, we investigated the transcriptome changes that occur in Katepwa as a consequence of ToxB treatment and compared these to our previously published transcriptome analysis of ToxA treatment of the same cultivar. Results of this current study further supports the hypothesis that plant defense responses are an active part of the complex compatible interaction between necrotrophic fungi and their host plants that precede the onset of cell death. Additionally, this comparative approach helps to define pathways that could be specific for individual effectors.

## Results

### ToxA and ToxB-induced Cell Death

**Figure 1:**
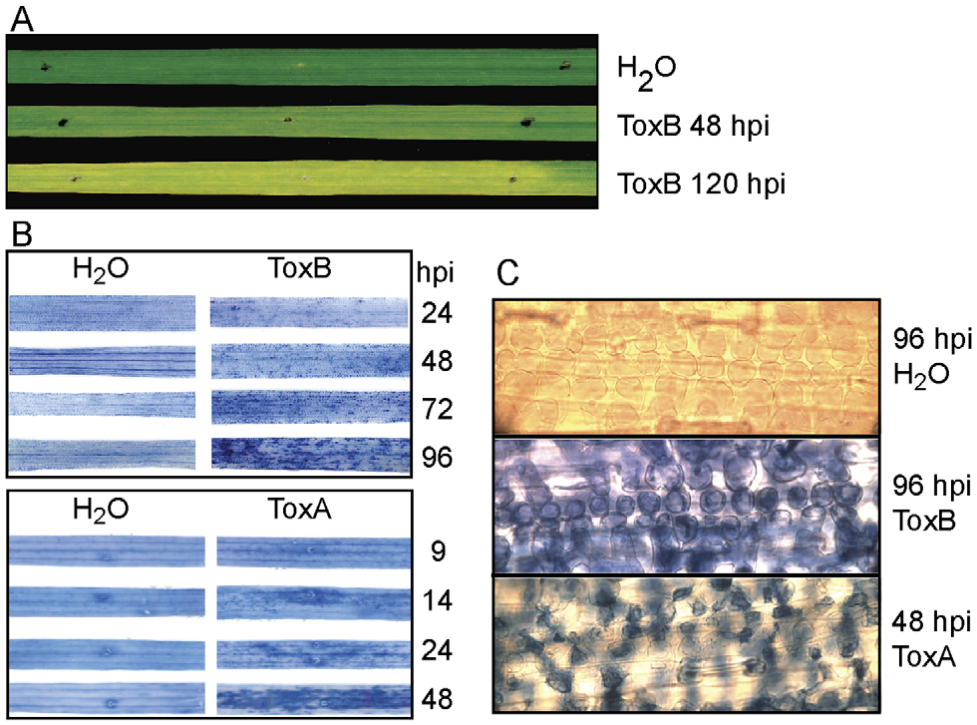
ToxB-induced symptom development and ToxB- and ToxA-induced cell death. (A) Bioassay of toxin sensitive Katepwa leaves infiltrated with either H_2_O (control) or ToxB. Black dots demarcate treatment zone. (B) Leaves were infiltrated with H_2_O, ToxB, or ToxA and stained with trypan blue. Leaves were collected at the indicated hours post infiltration (hpi). (C) Light microscopy of leaves infiltrated with H_2_O, ToxB, or ToxA and stained with trypan blue.

ToxA and ToxB treatment of the wheat cultivar Katepwa results in necrosis and chlorosis respectively. Necrosis or cell death in ToxA-treated leaves is easily visualized without additional staining of the tissue and first signs are detectable as early as 14 hours post-infiltration (hpi) with complete symptom development at 48 hpi [44]. In contrast, ToxB-induced chlorosis became visible at 48 hpi, intensified over 5 days and displayed less obvious signs of cell death (Figure 1A). To compare the progression of cell death in these treatments over time Katepwa leaves were infiltrated with ToxA, ToxB or water and treated with the vital stain, Trypan blue (Figure 1B). The initial differences in staining between water and toxin treatments were observed at 9 hpi for ToxA-treated (Figure 1B, bottom panel) and 24 hpi for ToxB-treated leaves (Figure 1B, top panel) and these differences increased over time. At 48 hpi, most of the ToxA-infiltrated leaf area was stained, whereas ToxB-treated leaves did not reach a similar level of staining, even at 96 hpi. Water-infiltrated control leaves displayed only background staining (Figure 1B). Because tissue collapse was apparent in ToxA-treated [44], but not ToxB-treated leaves (Figure 1A) we examined microscopically the morphology of the stained cells in both treatments at 48 and 96 hpi, respectively. At 48 hpi ToxA-infiltrated leaf tissue was intensely stained and the cells were deformed and collapsed with apparent gaps between cells (Figure 1C, bottom panel). In contrast, in ToxB-treated tissue, intensely stained cells still maintained their shape at 96 hpi (Figure 1C, middle panel).

### ToxB- and ToxA-Induced Changes on Global Gene Expression

The transcriptome changes that precede symptom development in ToxA-treated susceptible (Katepwa) leaves were previously examined [44]. To obtain information on ToxB-induced transcriptome changes, transcriptional profiles of ToxB and control leaves were compared. Because symptoms develop later in ToxB-treated leaves, in addition to the early time points of 3, 9 and 14 hpi which were analyzed in the ToxA experiment, the time course of this experiment was extended to 24 and 48 hpi. This allowed us to perform a comparative analysis of ToxB- and ToxA-triggered responses that precede the onset of symptom development. As with the ToxA analysis, the Affymetrix GeneChip® Wheat Genome Array was employed to obtain a global overview of differences in mRNA expression levels between control and ToxB-treated leaves. The previously obtained annotations [44] were used and supplemented with GO annotations published on the Affymetrix website (Table S1). The same four statistical methods were used to produce a high confidence annotated dataset for genes regulated under ToxB treatment (Figure S1A and B, Table S2). To validate the microarray data, RT-PCR was performed on selected probesets representative of gene groups discussed below. The overall pattern of gene expression was consistent with the microarray expression profile for each of the selected probesets (Figure S2).

**Figure 2:**
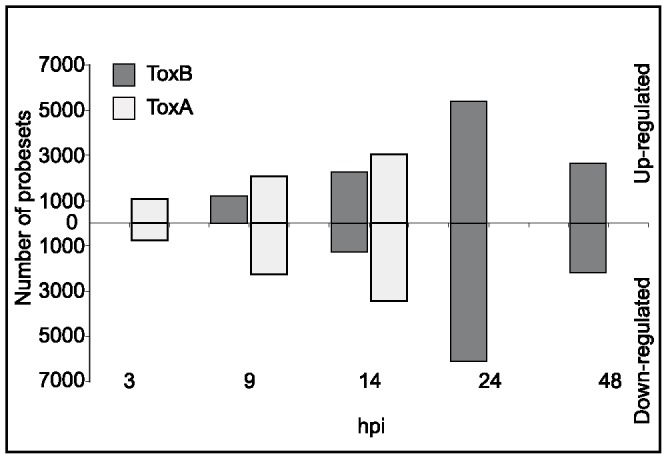
Comparison between ToxB- and ToxA-induced regulation of probesets in sensitive Katepwa leaves over time. Bar graph represents the number of statistically significant up- or down-regulated probesets for ToxA (white) and ToxB (light grey) as compared to H_2_O-infiltrated control at indicated hours post infiltration (hpi).

Based on the number of probesets that displayed differential mRNA expression levels when compared to the control, we concluded that ToxB treatment of sensitive wheat, like ToxA treatment of the same cultivar, induces massive transcriptional reprogramming. However, compared to ToxA treatment, these responses were delayed (Figure 2). Whereas ToxA induces significant changes in transcript levels at 3 hpi, no statistically significant ToxB-induced changes in mRNA levels were observed at this time point. At 9 hpi, more than 1000 probesets demonstrated higher mRNA levels (up-regulated) in response to ToxB; however, probesets with levels of mRNA lower than those of the H_2_O control (down-regulated) were not statistically significant at this time point. Similar to ToxA, the number of up- and down-regulated probesets increased over time in ToxB-treated leaves. Coincident with the first appearance of ToxB-induced chlorosis (Figure 1A), the total number of differentially expressed probesets drastically decreased at 48 hpi (Figure 2). This is in contrast to the ToxA responses when the first symptom development coincided with increasing changes in differential expression.

Gene Ontology (GO) term enrichment analysis [44], [46]–[48] previously used for ToxA data analysis was performed and a list of categories that were over-represented (high z-scores) in up- and down-regulated data sets for ToxB treatment was obtained (Table S3). At 9, 14 and 24 hpi the over-represented up-regulated categories included various kinases and the L-phenylalanine catabolic processes. Cell wall catabolic processes, chitinase activity, defense response to fungi, ATP binding, sugar binding, and calcium ion binding were also over-represented up-regulated categories at these time points. By 48 hpi, the groups described above no longer were over-represented; instead, up-regulated categories included transaminase and citrate synthase activity, intracellular signaling cascade, and cellular carbohydrate metabolic processes. At 14 hpi the most over-represented amongst the down-regulated groups were cation transport, photosynthesis, and lipid transport and by 24 hpi were components of the translation machinery, including structural constituents of ribosome and the “de novo” inosine monophosphate (IMP) biosynthetic process that is responsible for purine biosynthesis. At 48 hpi, components of photosynthetic processes were the most over-represented down-regulated group.

### Comparison of ToxB- and ToxA-Induced Effects on Specific Gene Families and Pathways

**Figure 3:**
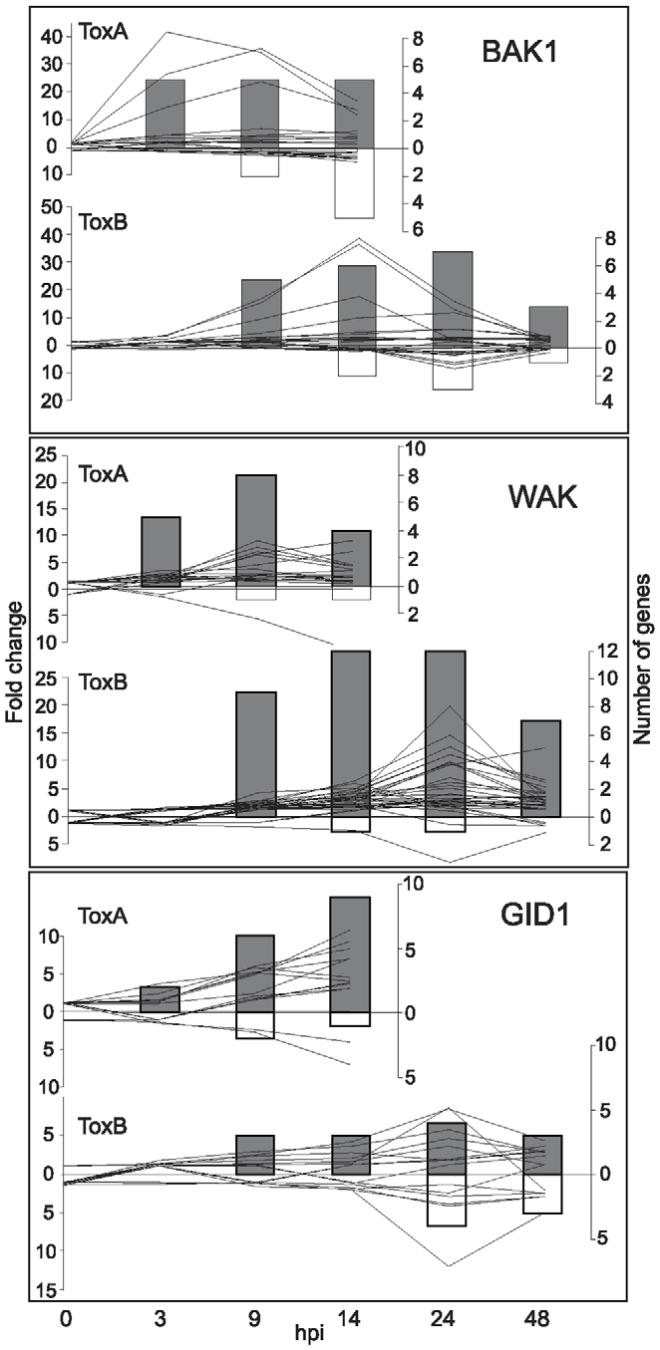
ToxB- and ToxA-induced regulation of receptor and receptor-associated families over time. Differences between control and toxin treatment presented as: line graphs for fold change (left axis) and bar graphs for the number of genes (right axis) that are significantly up- (lines above zero and grey bars) or down-regulated (lines below zero and white bars) at indicated hours post infiltration (hpi) (bottom axis). Receptor family names are to the top right of each graph, and include: brassinosteroid-associated receptor kinase (BAK1), gibberellin (GID1), and wall-associated kinase (WAK).

**Figure 4:**
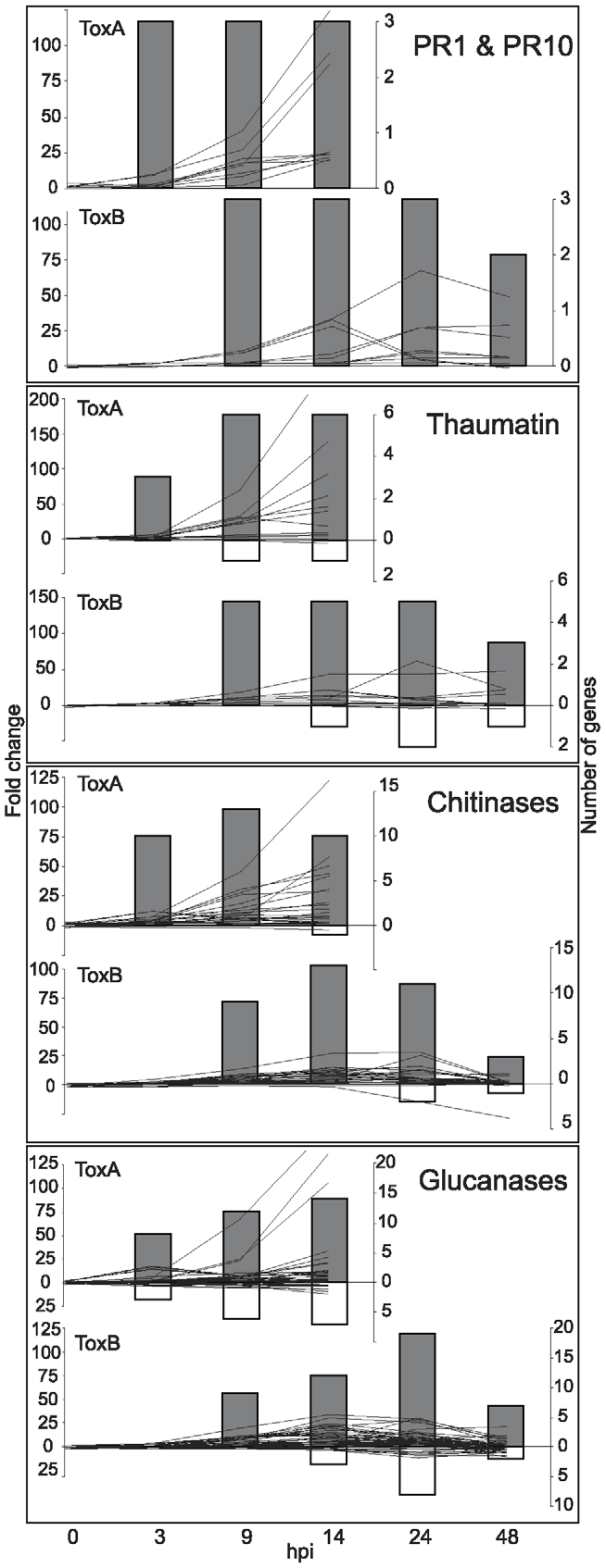
ToxB- and ToxA-induced regulation of groups of pathogenesis-related (PR) genes over time. Differences between control and toxin-treatment presented as: line graphs for fold change (left axis) and bar graphs for the number of genes (right axis) that are significantly up- (lines above zero and grey bars) or down-regulated (lines below zero and white bars) at indicated hours post infiltration (hpi) (bottom axis). PR genes family names are to the top right of each graph.

**Figure 5:**
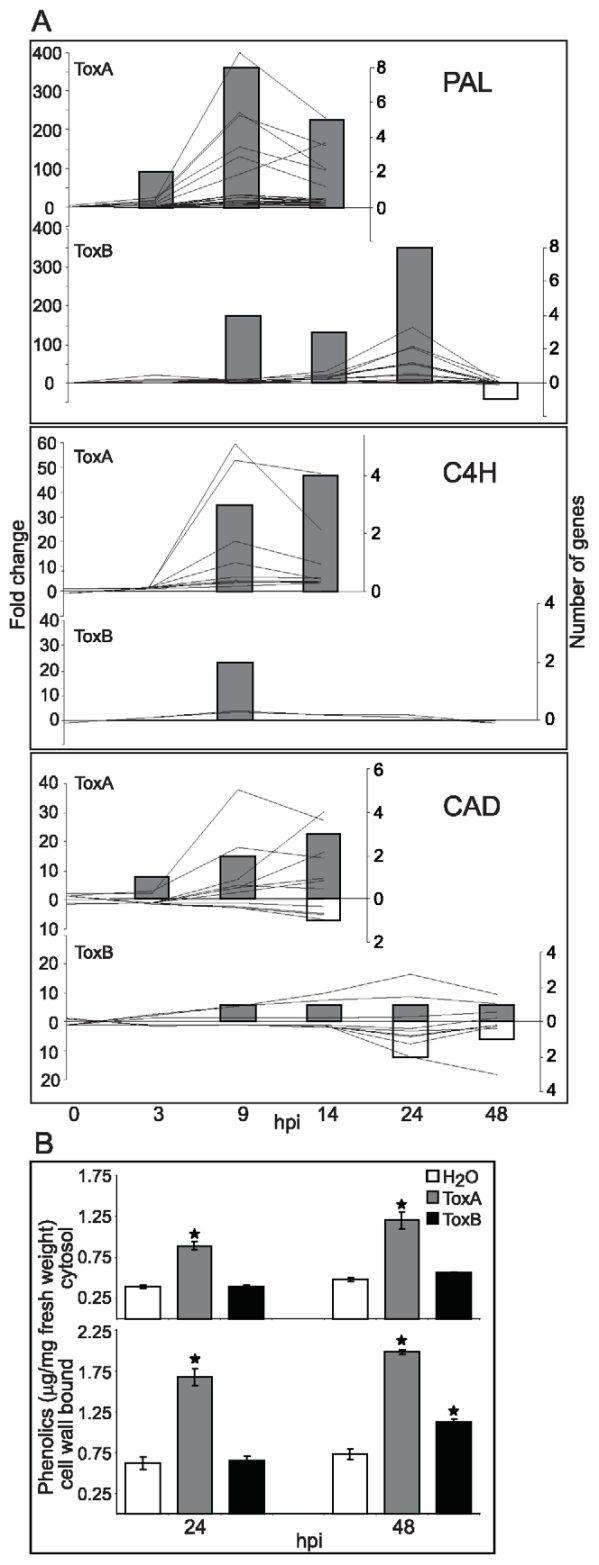
ToxB- and ToxA-induced activation of phenylpropanoid pathway. (A) Differences between control and toxin treatment presented as: line graphs for fold change (left axis) and bar graphs for the number of genes (right axis) that are significantly up- (lines above zero and grey bars) or down-regulated (lines below zero and white bars) at indicated hours post infiltration (hpi) (bottom axis). Gene family names are to the top right of each graph, and include: phenylalanine ammonia lyase (PAL), trans-cinnamate 4-monooxygenase (C4H), and cinnamyl alcohol dehydrogenase (CAD). (B) Accumulation of cytosolic and cell wall-bound phenolic compounds in leaves infiltrated with either H_2_O, ToxB, or ToxA at 24 and 48 hpi. Data represent mean and standard error of five biological replicates. (*) indicates means that are statistically significant from H_2_O treatment at each time point as determined by Student’s t-test (p<0.05).

**Figure 6:**
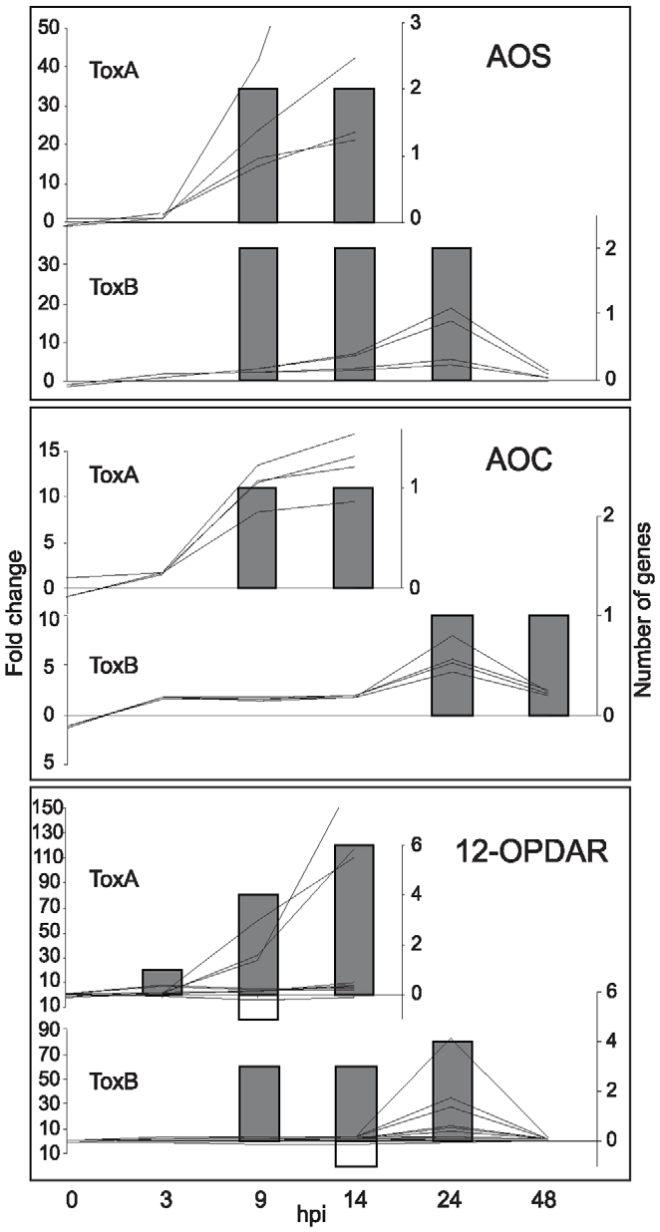
ToxB- and ToxA-induced regulation of genes involved in jasmonic acid biosynthesis. Differences between control and toxin treatment presented as: line graphs for fold change (left axis) and bar graphs for the number of genes (right axis) that are significantly up- (lines above zero and grey bars) or down-regulated (lines below zero and white bars) at indicated hours post infiltration (hpi) (bottom axis). Gene family names are to the right of each graph and include: allene oxide synthase (AOS), allene oxide cyclase (AOC), and 12-oxyphytodienoate reductase (12-OPDAR).

**Figure 7:**
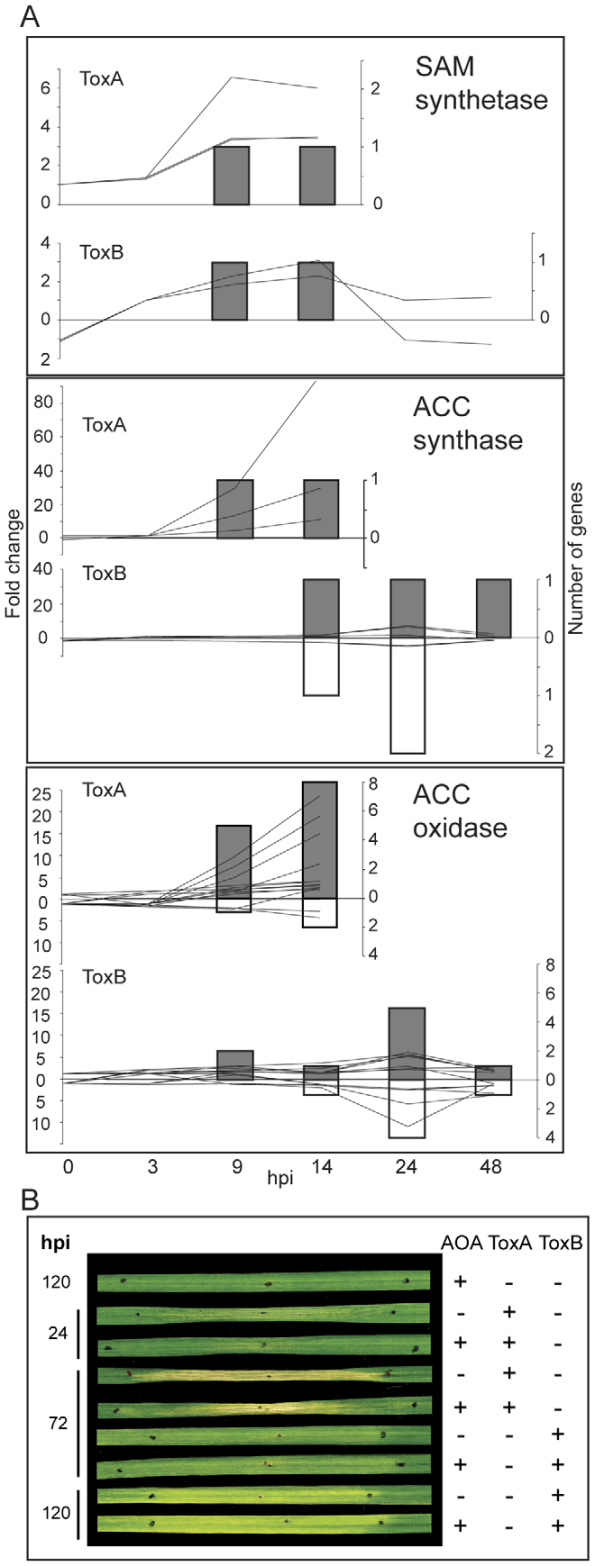
ToxB- and ToxA-induced regulation of genes involved in ethylene biosynthesis and the effect of an ethylene synthesis inhibitor on HST-induced symptom development. (A) Differences between control and toxin treatment presented as: line graphs for fold change (left axis) and bar graphs for the number of genes (right axis) that are significantly up- (lines above zero and grey bars) or down-regulated (lines below zero and white bars) at indicated hours post infiltration (hpi) (bottom axis). Gene family names are to the top right of each graph, and include: S-adenosylmethionine (SAM) synthetase, 1-aminocyclopropane-1-carboxylate (ACC) synthase, and ACC oxidase. (B) Bioassay of sensitive Katepwa leaves treated (+) or untreated (−) with either ToxA, or ToxB and/or the ethylene synthesis inhibitor aminooxyacetic acid (AOA). Black dots demarcate treatment zone. Leaves were collected at the indicated hpi.

**Figure 8:**
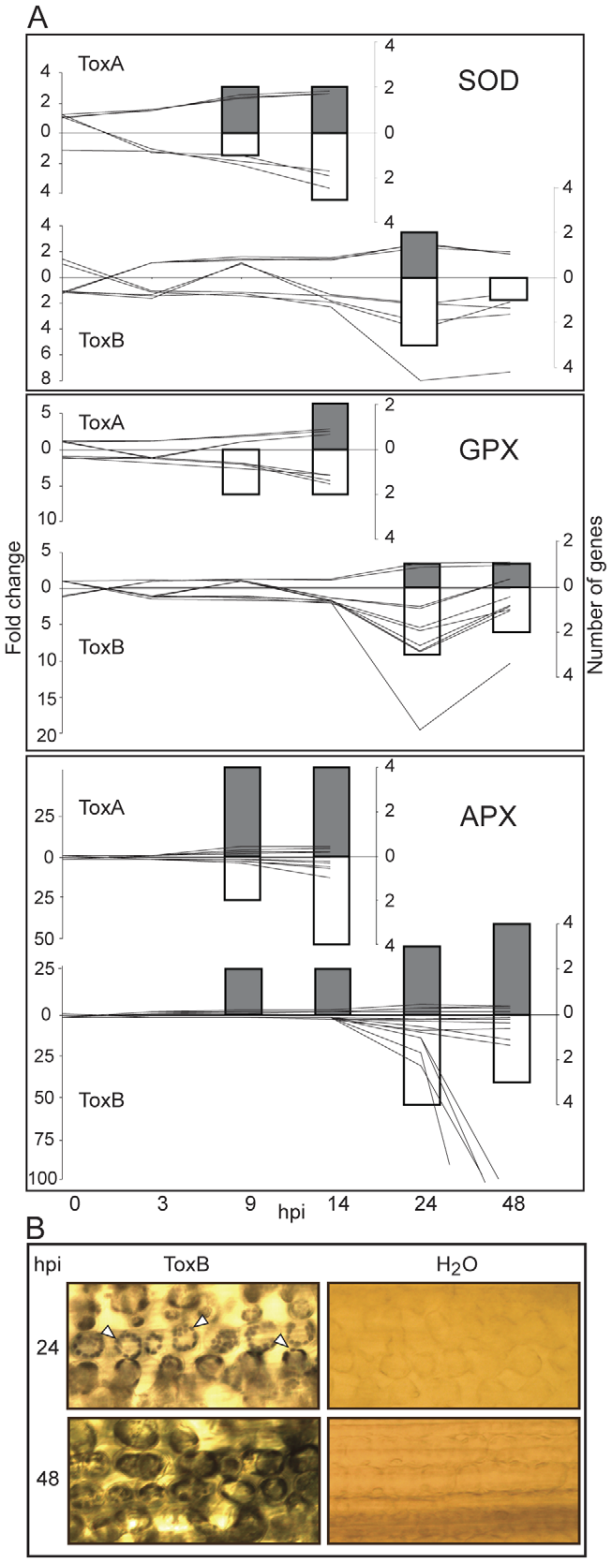
ToxB- and ToxA-induced regulation of genes involved in oxidative stress and the effect of ToxB on ROS accumulation. (A) Differences between control and toxin-treatment presented as: line graphs for fold change (left axis) and bar graphs for the number of genes (right axis) that are significantly up- (lines above zero and grey bars) or down-regulated (lines below zero and white bars) at indicated hours post infiltration (hpi) (bottom axis). The gene family names are to the top right of each graph, and include: superoxide dismutase (SOD), glutathione peroxidase (GPX), and ascorbate peroxidase (APX). (B) Light microscopy of leaves infiltrated with either H_2_O or ToxB and stained with nitroblue tetrazolium at 24 and 48 hpi. Arrowheads indicate examples of NBT stain associated with chloroplasts.

**Figure 9:**
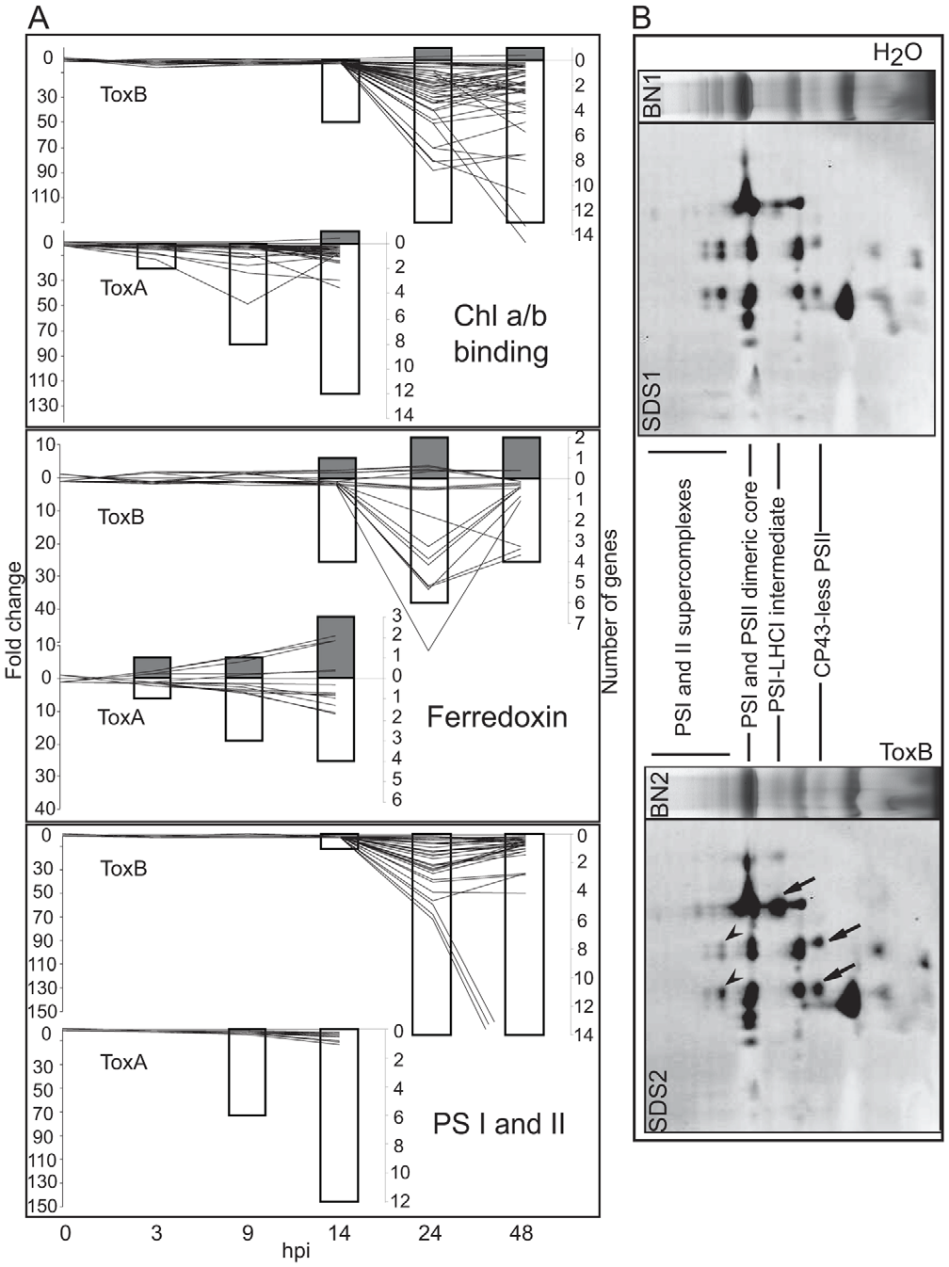
ToxB- and ToxA-induced regulation of genes involved in photosynthesis and the effect of ToxB on thylakoid proteins. (A) Differences between control and toxin treatment presented as: line graphs for fold change (left axis) and bar graphs for the number of genes (right axis) that are significantly up- (lines above zero and grey bars) or down-regulated (lines below zero and white bars) at indicated hours post infiltration (hpi) (bottom axis). Gene family names are to the bottom right of each graph, and include: photosystem I and II (PSI and II) and chlorophyll a and b (chl a/b). (B) Blue native-gel (BN1 and 2) electrophoresis (top panel) followed by SDS-PAGE (SDS1 and 2) of thylakoid fraction from H_2_O- or ToxB- treated leaves collected at 48 hpi. Arrows point to the complexes that are decreased (arrowheads) and increased (arrows) due to ToxB treatment. Protein content of these complexes is described in Manning et. al. 2009 [26].

**Figure 10:**
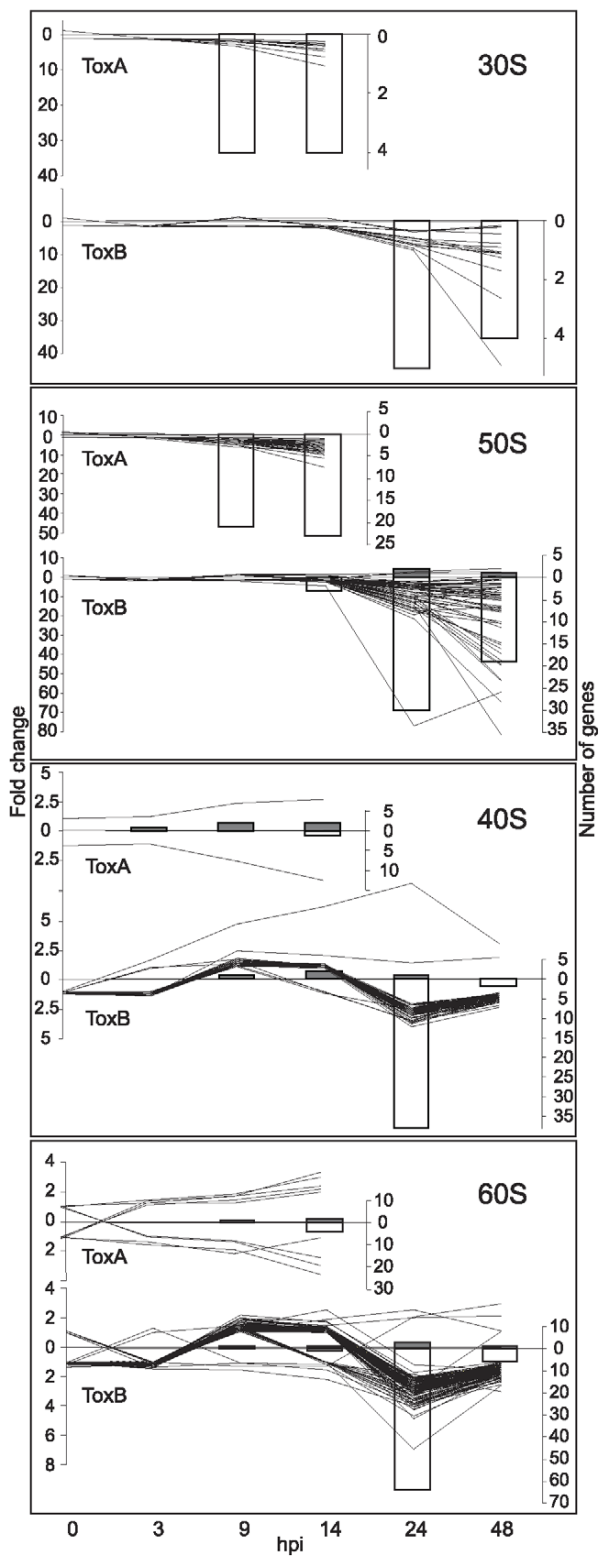
ToxB- and ToxA-induced regulation of ribosomal subunit genes. Differences between control and toxin-treatment presented as: line graphs for fold change (left axis) and bar graphs for the number of genes (right axis) that are significantly up- (lines above zero and grey bars) or down-regulated (lines below zero and white bars) at indicated hours post infiltration (hpi) (bottom axis). Gene family names are to the right of each graph.

To determine whether there are common and/or diverse mechanisms that govern plant susceptibility in response to different host-selective toxins, several groups of gene families that displayed transcriptional changes in response to ToxB were compared to the previously reported ToxA-dependent plant responses [44]. The transcriptional changes for the gene families discussed below are presented in Table S4. To have a more comprehensive comparison of transcriptome changes, both the number of genes and fold change patterns (see Table S4, columns Rice_HIT and Fold change, respectively) for their corresponding probesets are presented for both toxin treatments (Figures 3, 4, 5, 6, 7, 8, 9, 10).

#### Receptor kinases

Diverse groups of plant receptors present at the cell surface and/or inside cells are important components not only to detect environmental stresses, but also to initiate signal transduction cascades that lead to metabolic changes in the plant [49]. These changes ultimately determine the specific responses that the plant, as a host, will generate when attacked by a pathogen. The GO term enrichment analysis suggested that protein kinases were among the first groups of genes to respond to ToxB treatment. Thirty five percent of all probesets that represent a variety of receptors and receptor kinases were significantly up- or down-regulated in response to ToxB treatment. Similar to ToxA treatment these included ATP binding protein, lectin-like receptor kinases and glutamate receptor as well as receptor-like kinases (RLK) including leucine-rich repeat (LRR) receptor protein kinases and serine/threonine (Ser/Thr) protein kinases that are known to play a role in effector triggered immunity (ETI) (Table S5). Figure 3 illustrates transcriptional changes in response to the toxins of the three different types of receptors; brassinosteroid associated receptor-like kinase (BAK1) and wall-associated kinases (WAK) that represent a big group of receptor-like kinases, and gibberellin receptor (GID1). Despite their diversity, receptors were affected by both ToxA and ToxB treatments and were predominantly up-regulated, although fold change patterns showed differences in the time when maximal changes occurred. In both treatments BAK1 was up-regulated earlier than WAK and GID1. However, its interacting partner BRI1 receptor was found to be up-regulated only in ToxB-treated leaves (Table S4). The expression of WAK family genes showed a slightly higher fold change in ToxB- compared to ToxA-treated leaves (Figure 3). GID1 family gene expression patterns differed between the two treatments; maximum fold change in ToxB-treated leaves occurred prior to symptom development, whereas in ToxA-treated leaves there was a continuous increase in transcript level up to the time of symptom development at 14 hpi. Additionally, the number of up-regulated genes in ToxA was greater than in the ToxB experiment.

The unique structure of RLKs that contain cell surface receptor, transmembrane, and intracellular kinase domains allows these receptors to trigger signal transduction cascades in response to biotic and abiotic stresses. Like ToxA, ToxB treatment resulted in an increase of the mRNA levels for genes involved in calcium-dependent signaling pathways, ie. calmodulin-like and calcineurin-B-like proteins, calcium-dependent as well as mitogen-activated protein kinases (CDPK and MAPK) (Table S5). Transcription factors known to be involved in resistance responses, for instance WRKY, MYB, EREBP (ethylene-responsive element binding protein), and DRE (dehydration-responsive element), were also affected in both toxin treatments (Table S5).

#### Pathogenesis-related (PR) proteins

GO enrichment analysis indicated that defense response genes are involved not only in ToxA- but also in ToxB-induced plant responses. Genes such as *PR1* and *PR10*, thaumatin, chitinases and glucanases were found to be significantly up-regulated (Figure 4). Although, the number of genes regulated due to ToxA or ToxB treatment was similar for the different classes of PR genes, the first significant transcriptional changes detected in ToxA-treated leaves were at 3 hpi, while the ToxB-triggered transcriptional changes were detected consistently later at 9 hpi. The overall fold changes were higher in ToxA- vs. ToxB-treated leaves in all presented groups, but particularly in the chitinases. Additionally, expression levels increased until the point of onset of symptom development in ToxA-treated leaves, whereas in ToxB-treated leaves, expression levels peaked and then decreased prior to symptom development.

#### Phenylpropanoid pathway and cell wall modification enzymes

The phenylpropanoid pathway is responsible for the production of important secondary metabolites that can function as structural and signaling molecules involved in direct defense [50], [51]. Phenylalanine ammonia lyase (PAL), the key enzyme of this pathway, was an over-represented GO category at 9 hpi for ToxB-treated leaves. The transcript levels for this group of genes were significantly increased, with a maximum of 145 fold increase at 24 hpi (Figure 5A). Although, the same overall number of PAL genes was up-regulated in both toxin treatments, ToxA-induced maximum changes were observed already at 9 hpi with up to a 400 fold increase. The transcript levels for the second enzyme in phenylpropanoid pathway cinnamate-4-hydroxylase (C4H), that leads to the formation of phenolic compounds was increased up to 60 fold in ToxA-treated leaves, whereas very few probesets with considerably lower fold change were up-regulated in ToxB-treated leaves.

In order to evaluate if these transcriptional differences reflect accumulation of phenolic compounds, which we predicted would be greater in ToxA vs. ToxB treatment, cytosolic and cell wall-bound phenolics were extracted and quantified in control, ToxB-, and ToxA-treated leaves. In the ToxA treatment, maximum increase in levels of the transcript for PAL and C4H (Figure 5A) was followed by a significant accumulation of cytosolic and cell wall-bound phenolics (Figure 5B) at 24 hpi and 48 hpi. In contrast, the lower levels of transcript for the PAL and C4H enzymes (Figure 5A) in ToxB-treated leaves correlated with a small increase of only cell wall-bound phenolics at 48 hpi (Figure 5B).

The transcript levels for other enzymes in the PAL pathway, like 4-coumarate-CoA ligase (4CCoAL), caffeoyl-CoA O-methyltransferase (CCoAM), cinnamyl alcohol dehydrogenase (CAD) were also altered in response to ToxB (Table S5). Similar to PAL, the changes in expression of these genes in response to ToxB treatment occurred later in time and with a lower fold-change when compared to ToxA (Table S4). The majority of the genes/probesets for cinnamyl alcohol dehydrogenase (CAD), an enzyme leading to lignin production, were down-regulated in response to ToxB, whereas more probesets were up-regulated in response to ToxA treatment (Figure 5A).

Plant polygalacturonases (PGs) are involved in cell wall modification and control of cell growth and development as well as wound responses and plant-pathogen interactions [52], [53]. Oligosaccharides produced by PG activity induce production of polygalacturonase inhibitor protein (PGIP) involved in the defense response. As in the case of ToxA, the majority of PG precursor probesets and PGIP were up-regulated in response to ToxB (Table S5).

#### Jasmonic acid and ethylene biosynthesis pathways

The number of genes for the enzymes involved in the biosynthesis of jasmonic acid (JA) that were up-regulated was similar in response to ToxA and ToxB. Phospholipases (PL) and lipooxygenases (LOX) that represent a functionally diverse spectrum of genes, some of which may be involved in JA synthesis, showed up- or down-regulation (Table S5). In contrast, more specific enzymes in the pathway such as allene oxide synthase (AOS), allene oxide cyclase (AOC), and 12-oxophytodienoate reductase (12-OPDAR) displayed a more consistent trend (Figure 6). Again, the transcriptional changes in these genes occurred earlier in time and with higher fold change in ToxA- than in ToxB-treated leaves.

ToxA treatment results in up-regulation of the genes involved in the ethylene biosynthesis pathway [44]. Comparison of the transcriptional changes induced by ToxB revealed that S-adenosylmethionine (SAM) synthetase, an enzyme required for production of a precursor SAM in the biosynthesis of ethylene, polyamines, biotin, and nicotianamine in plants, was up-regulated in both ToxA and ToxB treatments (Figure 7A). However, the evaluation of the fold change patterns for 1-aminocyclopropane-1-carboxylase (ACC) oxidase and ACC synthase, two major enzymes specific to the ethylene biosynthesis pathway, showed a higher fold up-regulation in response to ToxA compared to ToxB, suggesting that ethylene biosynthesis is more active in ToxA-treated leaves. In order to examine if ethylene biosynthesis plays a greater role in ToxA than in ToxB symptom development we used an ethylene synthesis inhibitor, aminoacetic acid (AOA) that inhibits the ACC synthase step by preventing conversion of SAM to ACC which in turn is converted to ethylene by ACC oxidase. The bioassay results shown in Figure 7B demonstrate that infiltration of 10 µM AOA alone did not cause morphological changes in Katepwa leaves. Symptoms on leaves treated with ToxA+AOA were observed at 24 hpi, and were reduced significantly compared to ToxA treatment alone (15±5% vs. 28±6% damage, p<0.01). At 72 hpi, symptoms in AOA+ToxA treated leaves although intense, still do not extend over the whole infiltration zone like in ToxA-treated leaves (69±13% vs. 91±5% damage, p<0.01). Similar experiments with ToxB and ToxB+AOA (Figure 7B) did not result in a noticeable reduction of ToxB-induced chlorosis at either 72 (9±3% vs. 10±5% damage) or 120 hpi (71±7% vs. 66±14% damage).

#### ToxB-induced ROS accumulation and its effect on energy dependent processes

Reactive oxygen species (ROS) are a byproduct of energy-dependent processes, such as photosynthesis and respiration [54]. The main organelles that generate ROS in plants are mitochondria, chloroplasts and peroxisomes, all of which contain an extensive array of oxidases. Microarray data showed increases in transcript levels of several oxidases whose activity results in generation of extracellular H_2_O_2_, i.e., oxalate and reticuline oxidase, upon ToxA and ToxB treatments, and sarcosine oxidases, only in ToxB-treated leaves (Table S5). Given that the accumulation of ROS in a plant cell is harmful, detoxifying enzymes, such as superoxide dismutase (SOD), catalase, glutathione (GPX) and ascorbate peroxidases (APX), facilitate the maintenance of low levels of ROS in cells. In ToxB-treated leaves, genes/probesets for SOD and GPX showed differential expression at 24 hpi, whereas the changes in transcript levels for APX genes/probesets were detected already at 9 hpi (Figure 8A). Interestingly, in both treatments up- or down-regulation of the probesets depended on organellar localization evident by down-regulation of the genes associated with chloroplasts and the up-regulation of cytosolic genes. Additionally, catalase transcript levels were affected and several up- and down-regulated probesets were present in response to ToxB, whereas only one probeset was down-regulated in ToxA treatment (Table S5). The majority of probesets for another important detoxifying enzyme, glutathione S-transferase, were up-regulated in both ToxB and ToxA-treated leaves (Table S5).

Accumulation of chloroplastic ROS was detected in ToxA-treated leaves stained with Nitroblue tetrazolium (NBT) [26]. The same method was used to test if ROS accumulation was, indeed, part of the ToxB mode-of-action. NBT staining was observed in ToxB-treated Katepwa leaves at 24 hpi and, like ToxA, the majority of the stain was associated with chloroplasts (Figure 8B, arrows). The intensity of the stain increased at 48 hpi; however, at this point, the localization of the stain was not restricted to the chloroplast.

As in the case of ToxA, photosynthesis was one of the over-represented down-regulated GO categories in the ToxB microarray analysis. The major components of the photosynthetic machinery including photosystem (PS) I and II, ferredoxin, and chlorophyll a/b were significantly down-regulated (Figure 9A). For some PSI and PSII probesets the down-regulation by ToxB treatment was extensive (in some cases >200 fold), and greater than the down-regulation by ToxA (<20 fold). In ToxB-treated leaves the majority of probesets reached a maximal change in expression levels at 24 hpi for each of these photosynthetic components. To ascertain if the decrease in transcript levels in ToxB treatment was reflected in the protein profile, thylakoids were isolated from control and ToxB-treated sensitive leaves at 24 and 48 hpi and examined by Blue Native (BN) gel followed by SDS-PAGE. The BN gels showed similar amounts of total thylakoid protein in control and ToxB treatment at 24 hpi (data not shown) and slight changes at 48 hpi: a decrease in PSI and II supercomplexes, and increase in PSI-LHCI (light-harvesting complex) intermediate and CP43-less PSII complexes (Figure 9B, BN1 and BN2). SDS-PAGE of 48 hpi samples showed that the intensities of the largest spots corresponding to the components of PSI and PSII supercomplexes were reduced (Figure 9B, arrowheads), whereas the intensities of the spots corresponding to the components of PSI-LHCI and CP43-less PSII intermediate complexes were increased (Figure 9B, arrow).

In addition to the changes in transcript levels for photosystem components, the set of probesets for chloroplast enzymes involved in the Calvin cycle were also affected by both toxin treatments. The majority of the probesets/genes that correspond to Ribulose-1,5-bisphosphate carboxylase/oxygenase (RuBisCO), fructose 1,6-bisphosphate aldolase and glyceraldehyde-3-phosphate dehydrogenase were down-regulated in response to both toxins. Transketolase and triosephosphate isomerase were down-regulated only in ToxB-treated leaves at 24 and 48 hpi (Table S5). In contrast, probesets/genes that correspond to enzymes of the Krebs cycle, responsible for ATP synthesis by generating NADH and FADH_2_ in the mitochondrial matrix, such as citrate synthase, isocitrate, malate and pyruvate dehydrogenases were up-regulated in response to ToxB and/or ToxA (Table S5).

#### Protein biosynthesis and degradation

According to GO term enrichment analysis, both HSTs had a negative regulatory effect on the transcription of ribosomal components (Table S3). However, the level of down-regulation, and which subunits were affected differed between treatments. The number of down-regulated genes for chloroplast associated ribosomal subunits (30S and 50S) was similar for both toxin treatments but with the greater fold change in ToxB-treated leaves (Figure 10). In contrast, the number of down-regulated genes encoding cytosolic subunits (40S and 60S) was significantly higher in ToxB treatment (37 and 65, respectively) (Figure 10) compared to ToxA treatment (1 and 5, respectively).

Both ToxA and ToxB also had an effect on transcript levels of components of the ubiquitin/26S proteosome system. Three enzymes responsible for ubiquitination processes such as ubiquitin-activating (E1), -conjugating (E2), ligase (E3) and an additional conjugating enzyme (E4), were differentially expressed with more up-regulated probesets in ToxB treatment (Table S5). Additionally, a considerably greater number of 26S proteosome-related probesets/genes were up-regulated in ToxB-treated leaves.

## Discussion

To explore and characterize the underlying mechanisms of susceptibility to a necrotrophic pathogen, we investigated plant responses to the HST ToxB, and compared them to the responses evoked in leaves treated with the HST ToxA [44]. Though these two HSTs require different sensitivity loci, the availability of a wheat cultivar sensitive to both toxins, and an equivalent microarray experimental design, made this comparison feasible and robust.

Symptoms produced by ToxA (necrosis) and ToxB (chlorosis) are not only visually distinct but also develop at a different rate. In both toxin treatments, cell death as assessed by vital stain, was detected before symptoms became apparent, i.e. 24 hpi in ToxB-treated and 9 hpi in ToxA-treated leaves (Figure 1B). Additionally, the difference in morphology of the stained cells suggests that the tissue collapse in ToxA-treated cells is likely due to cellular disruption and formation of intercellular gaps, a phenomenon that does not appear to occur in ToxB-treated leaves even with advanced chlorotic symptoms.

Comparative transcriptional analyses of the responses triggered by ToxA and ToxB confirmed that the later onset of symptom development in ToxB-compared to ToxA-treated leaves correlated with later transcriptional responses to the toxin. The first statistically significant changes in transcript levels were observed at 9 hpi in ToxB-treated leaves (Figure 2) vs. 3 hpi in ToxA-treated leaves. It is possible that this temporal shift in the transcriptional responses is the result of differences in the perception of these toxins by the plant cell and/or the site-of-action. We have accumulated evidence that ToxA binds to a high affinity receptor on sensitive plants. Furthermore, detection of ToxA in the cytoplasm and chloroplasts of ToxA-sensitive cells, in combination with the cytoplasmic localization of the *Tsn1* gene required for ToxA-induced necrosis, suggest an intracellular site-of-action for ToxA [22], [23], [38]. While evidence for the site-of-action of ToxB is still under evaluation, structural and biochemical data suggest that it likely functions in the apoplast [30]. In comparison to ToxA, an apoplastic site-of-action for ToxB and/or a low affinity receptor could explain the increased time required for the plant to respond.

Several examples of intracellular plant receptors that are encoded by sensitivity-conferring genes have been described. In *Arabidopsis thaliana*, sensitivity to the toxin victorin produced by *Cochliobolus victoriae*, is dictated by *LOV1*, a member of the NBS-LRR resistance gene family [41]. In sorghum, the *Pc* gene, which confers sensitivity to Peritoxin (Pc-toxin) produced by *Periconia circinata*, represents another example of a toxin sensitivity-conferring gene that encodes NBS-LRR domains [42]. In addition, the recent cloning of *Tsn1* revealed that this gene encodes S/TPK and NBS-LRR domains, which are characteristic of resistance genes [38]. The prospect of *Tsn1* acting as a resistance gene can explain how ToxA can elicit defense responses similar to those associated with resistance. These responses included up-regulation of WRKY transcription factors, PR genes; genes associated with the phenylpropanoid, ethylene and jasmonic acid (JA) pathways [43], [44]. Interestingly, similar defense responses are activated by ToxB treatment, suggesting that the induction of plant defense is a common response to both toxins. This is in stark contrast to the effectors that induce host susceptibility by suppressing defense [55]. Our findings support the model that susceptibility and resistance have overlapping signaling pathways and responses [2], mediated by a similar class of intracellular receptors, and raise the question whether the ToxB sensitivity locus, *Tsc2*, will encode a gene similar to R genes.

**Figure 11:**
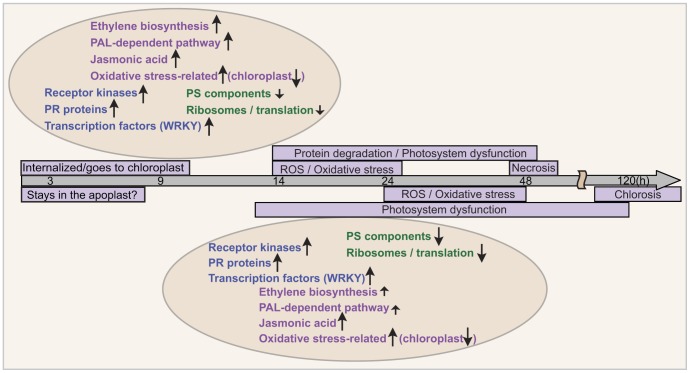
A timeline of transcriptional and biochemical changes in response to ToxA or ToxB treatment of sensitive wheat. Numbers in the grey arrow indicate the times (h = hours post infiltration) at which changes have been observed. Light purple boxes contain descriptions of biochemical changes and tan ovals contain descriptions of transcriptional changes. Arrows indicate up- or down- regulation and the size of the arrow reflects differences in amplitude between shared responses to ToxA and ToxB treatments.

Membrane-localized as well as intracellular receptors play a critical role in the recognition and signal transduction in response to environmental stimuli. For example, RLKs are known to play a role in effector triggered immunity (ETI). BAK1 plays a role not only in BR (brassinosteroid) signaling through interaction with brassinosteroid receptor BRI1 but has also been implicated in PTI (PAMP-triggered immunity) [56]. The hormone receptor GID1 [57], [58], that is a major component of gibberellin signaling is involved in plant development and growth as well as the stress response [54]. WAK family genes have been shown to be involved in cell expansion, pathogen resistance, and heavy-metal stress tolerance in *Arabidopsis thaliana* [59], [60]. Analysis of *Arabidopsis thaliana* expression data established that hundreds of RLK/Pelles are up-regulated by biotic stress [61]. These authors proposed that the massive up-regulation of normally constitutively expressed RLK/Pelles is necessary in order to detect additional highly variable molecular patterns (MAMPs) associated with specific pathogens. By expressing a large number of potential receptors, a plant can activate a strong defense response initiated by the interaction of one or more up-regulated receptors with its corresponding MAMP. The remarkable number of diverse groups of receptors up-regulated in response to both ToxA and ToxB, including RLKs and hormone receptors, suggests that these HSTs use pathways that overlap with those utilized by MAMPs. It is unclear though, how many of the up-regulated receptors are translated and actually involved in triggering downstream signaling events. The ubiquitin/26S proteosome system is connected to several transduction pathways as it plays an important regulatory role in signaling by plant hormones especially auxin, JA and gibberellin [62], [63]. This connection could explain the fact that along with up-regulation of hormone receptors we found an increase in the mRNA levels of proteins involved in ubiquitination in both toxin treatments. However, expression of the components of the 26S proteasome is higher in ToxB vs. ToxA treatment, suggesting that protein degradation is part of the response only to ToxB, but not ToxA treatment where cell death may be too rapid for the up-regulation of the 26S proteosomes to occur.

The activation of the phenylpropanoid pathway is an important defense response, crucial for the reinforcement of cell walls and the production of lignin and antimicrobial metabolites, such as flavonoids and phytoalexins [64]. While some enzymes in this pathway, PAL, 4CCoAL and 4CCoAM were induced similarly by both toxins, others like C4H, an enzyme involved in the production of phenolic compounds, and CAD, the enzyme necessary for lignin production, showed differences in expression (Figure 6 and Table S4). The up-regulation of PAL in response to both toxins is not surprising, as the activation of this enzyme has been reported in several pathosystems as well as in response to toxins [41], [44], [65]. Differences found between responses to ToxA and ToxB in expression levels of C4H that correlate well with levels of phenolic compound accumulation and also CAD expression levels suggest that lignin production is minor in ToxB-treated leaves compare to ToxA. Lignin production would most likely restrict pathogen growth if lignification occurs prior to rapid cell death in ToxA-treated leaves. The deficiency in lignification in response to ToxB could allow for continued growth of ToxB expressing fungal isolates while it slowly induces cell death.

Ethylene-dependent defense responses to necrotrophic pathogens [66] as well as the accumulation of ethylene in response to several toxins, for example, victorin, fusicoccin and AAL-toxin [2], [65], [67] are well-documented. Both ToxA and ToxB treatments, lead to changes in the mRNA level of the enzymes involved in the ethylene biosynthesis pathway. AOA considerably delayed symptom development in ToxA- but not in ToxB-treated leaves, suggesting that ethylene may play a role in accelerated symptom development in ToxA-treated leaves. These results are consistent with the suggested role of ethylene as a modulator of cell death [68] and amplifier of ROS accumulation [69]. The positive regulation that has been proposed to occur between ethylene and ROS is of interest, as ToxA-induced cell death has been shown to be mediated by the production of ROS [26].

Similarly, the JA pathway is up-regulated in response to both toxins. Fungal pathogens and elicitors are known to induce accumulation of JA in plant cells [70]–[72] and it can play a role in plant development, senescence, wound responses and defense [73]. JA accumulation inhibits the synthesis of photosynthetic proteins such as the ribulose-1,5-bisphosphate carboxylase/oxygenase large subunit, causes mitochondrial ROS production, and prompts chlorophyll breakdown by weakening the major light-harvesting chlorophyll a/b binding protein complexes associated with photosystem I and II [73]–[77]. Because chlorophyll a/b binding protein and Ribulose-1,5-bisphosphate carboxylase/oxygenase are down-regulated in both treatments and there is a breakdown of chlorophyll upon ToxB treatment [33], it is possible that the accumulation of JA may contribute to the mode-of-action of ToxB and ToxA.

An appropriate redox environment in plant cells is controlled by the coordinated function of the ROS producing pathways and detoxification mechanisms. Nonetheless, this balance can shift towards ROS accumulation under the presence of various biotic stresses, such as pathogen attack [78]. In host-pathogen interactions, ROS serve as signals to activate basal resistance and ETI [78], [79]; however, fine regulation is required to avoid a harmful accumulation of ROS that can result in enhanced susceptibility and cell death [80], [81]. As pathogenicity/virulence factors for necrotrophic pathogens, we might expect that HSTs would induce ROS accumulation to enhance susceptibility. Up-regulation of several oxidase genes involved in the formation of extracellular ROS speaks in support of extracellular ROS induction by both ToxA and ToxB. Additionally, NBT staining confirmed ROS production in both ToxA- [26] and ToxB-treated leaves and in both cases ROS accumulation was detected predominantly in chloroplasts (Figure 8B). Surprisingly, in both ToxA and ToxB treatments, this accumulation of ROS in chloroplasts was not accompanied by an up-regulation of detoxification enzymes that are localized to the chloroplast (Table S4). However, a wide spectrum of cytosolic enzymes involved in ROS detoxification and oxidative stress-coping mechanisms are up-regulated (Figure 8). Proteomics and metabolomics studies on the effects of ToxA and ToxB in sensitive leaves demonstrate an increase in protein activity of ascorbate peroxidase, and total peroxidases [34], differential abundance of ROS detoxifying proteins, and a decrease in photosynthetic proteins and perturbations in central carbon metabolism [82].

Comparative analysis of publically available transcriptome data that included twenty two different forms of biotic damage shows that down-regulation of photosynthesis-related genes is a common response to biotic stress and could be a compensatory reaction to allow the plant to defend itself from environmental stresses in support of up-regulation of defense-related genes [83]. Additionally, a computational analysis revealed that there are several common regulatory elements in the promoter regions of photosynthetic genes and some genes involved in sugar metabolism and ROS detoxification suggesting that transcriptional responses to biotic stress are highly coordinated [83]. Down-regulation of components of PSI, PSII, and chl a/b proteins by both ToxA and ToxB and the down-regulation of chloroplast ROS detoxification enzymes suggest that HSTs act similar to other biotic agents that regulate gene expression through common elements. It appears that in the case of both ToxA and ToxB treatment, while the host is down-regulating these chloroplast-localized genes in the hopes of ridding itself of the pathogen by the production of defense-related proteins, it is damaging itself by not up-regulating the enzymes that could prevent ROS accumulation in the chloroplasts.

ROS accumulation is thought to play a major role in the effect of ToxA on photosynthetic machinery and subsequent necrosis development. Changes in homeostasis of PSI and PSII components observed in the absence of ROS (dark incubation), were dramatically enhanced in the presence of ROS (24 h of light incubation), resulting in a decrease in total thylakoid protein content [26]. In fact, a reduction of total thylakoid protein content can be observed as early as 14 hours post ToxA treatment (unpublished data). The observed decrease in protein content could be explained by destruction of proteins due to oxidative damage and the inability to restore them due to down-regulation of transcripts that encode components of PSI, PSII and chl a/b. However, unlike treatment with ToxA and despite chloroplastic ROS accumulation and a decrease in transcripts for PSI, PSII and chl a/b proteins, there was no drastic decrease in total thylakoid protein in treatment with ToxB, only a slight reduction in PSI and PSII supercomplexes and an increase in PSI and PSII intermediate complexes at 48 hpi. One possible explanation for this difference is reduced protein turnover in the PS complexes of ToxB-treated leaves. Protein turnover may not be necessary due to decreased net rates of photosynthesis, which are detectable as early as 12 hpi in ToxB-treated leaves [34]. Additionally, during the 48 hours post toxin treatment ToxB-treated plants undergo two dark incubation periods where photosynthesis does not occur, alleviating the need for photosystem protein repair. The decrease in supercomplex formation and the increase in PSI and PSII intermediate complexes could be due to the decrease in transcript levels of some components required for assembly of these complexes. It was shown that absence of monomeric subunits CP26 and CP29 in light harvesting complexes reduces the stability of PSII-LHCII supercomplexes [84]. Although to a lesser extent than ToxA [26], ToxB nonetheless alters protein homeostasis of PSI and PSII.

In contrast to photosynthetic genes, there is an increase in transcript levels of genes encoding mitochondrial enzymes involved in the Krebs cycle for both toxin treatments that is also supported by proteomics study of the *Stagonospora nodorum* SnToxA [82]. This increase could indicate the cell’s attempt to fulfill metabolic and physiological demands and that the mitochondrial machinery tries to compensate for the loss of energy production in the chloroplast. It is also possible that massive down-regulation of genes for cytosolic ribosomal proteins in ToxB-treated leaves occurs to decrease efficiency of translational machinery in order to save energy. However, in ToxA-treated leaves genes for cytosolic ribosomal subunits are not massively down-regulated prior to symptom development. An earlier study [85] showed that the protein synthesis inhibitor cycloheximide prevented necrosis development and electrolyte leakage in ToxA-treated leaves suggesting that ToxA-induced necrosis requires active protein synthesis.

This comparative analysis of ToxA- and ToxB-induced host responses further supports the hypothesis that activation of host defense is an important part of a susceptible interaction between a necrotrophic pathogen and its host plant. Figure 11 summarizes processes that are initiated by the host following ToxA or ToxB treatments. Though activated defense responses are similar, ToxA induced transcriptional and biochemical responses and symptom development are faster and more robust compared to ToxB. The difference in the time of responses between toxins could be explained by differences in perception as well as by differences in activation of a particular process like ethylene biosynthesis. Slower responses and in some cases lower amplitudes in gene expression that lead to correspondingly lower biochemical changes in ToxB-treated leaves allows the plant to maintain homeostasis longer and therefore, result in slower and less severe symptom development. The chloroplast appears to be a common target for both toxins and is subject to accumulation of ROS and disruption of photosynthesis. Given that ROS can induce defense responses, it is possible that defense gene regulation in response to these toxins is also ROS dependent. Evaluation of the impact of these toxins on host gene expression in the absence of ROS would be valuable in addressing this issue.

## Materials and Methods

### Plant Growth and ToxA and ToxB Production

Genotypes of wheat cultivars used in this study were chosen based on their sensitivity (Katepwa) or insensitivity (Auburn) to ToxA and ToxB [17], [18], [21], [86], [87]. Plants were grown in a growth chamber under a cycle of 16 hours (h) of light at 22°C, and 8 h of darkness at 18°C. Native ToxA and ToxB were produced in liquid culture and purified to homogeneity by gel filtration chromatography followed by HPLC [18], [88]. Heterologously (het) expressed ToxA and ToxB were produced in *Pichia pastoris* and purified by affinity chromatography [23], [88]. Protein concentration was assessed by the Pierce BCA protein assay kit (Pierce, Rockford, IL, U.S.A.) with bovine serum albumin as a standard.

### Experimental Design and Analysis of the Microarray Data

Either ToxB (15 µM) or H_2_O was infiltrated into secondary leaves (8 leaves per treatment) of the ToxB-sensitive wheat cultivar Katepwa using a modified Hagborg device [89] and the infiltration zone was defined by black dots. A 4-cm leaf area surrounding the infiltration center point was collected at 0, 3, 9, 14, 24 and 48 hours post infiltration (hpi), ground in liquid nitrogen and stored at −80°C for later RNA isolation. Three biological replicates were performed.

Total RNA isolation and quality control was performed as described previously [44]. RNA concentration was measured on a NanoDrop® ND-1000 UV-Vis spectrophotometer (NanoDrop Technologies, Wilmington, DE) with a resulting concentration in the range of 600–1250 ng/µl in 30 µl. RNA integrity screening, probe synthesis, hybridization and scanning were conducted by the Center for Genome Research and Biocomputing Core Laboratories at Oregon State University, Corvallis, OR. We utilized the Affymetrix GeneChip® Wheat Genome Array (Affymetrix, Santa Clara, CA, USA) that includes 61,127 probesets representing 55,052 transcripts of all 42 wheat chromosomes. Microarray data quality, normalization and identification of differentially expressed probesets was performed as described previously [44] and summarized in Figure S1A and B. One of the replicates for the 3 hpi of the ToxB treatment dataset exhibited higher probe intensities than the two other sets. Thus, to ensure accuracy of the analysis, an additional statistical analysis was conducted for that specific time point using only two replicates. This approach confirmed that the original analysis was valid, because only two probesets showed statistically significant change in expression at 3 hpi. Several probesets, which were differentially regulated in our previous microarray analysis, were successfully validated by reverse transcriptase (RT)-PCR [44]. A subset of these probesets was chosen to confirm and compare expression patterns derived from the current study. Probesets for genes of interest were chosen from those that represented various fold change ranges and expression patterns. Experiments were performed using two biological replicates and repeated at least two times, with similar results, for each probeset selected.

### Viability Staining and Inhibition of Ethylene Production

Viability staining was performed using Trypan blue stain as previously described [90] with some modifications. Briefly, secondary wheat leaves were infiltrated with either H_2_O, heterologously expressed 2 µM hetToxA or 20 µM hetToxB, and a 3 cm-leaf section around the infiltration zone was collected at the indicated hpi and boiled in staining solution for 1 min. Leaves were incubated for 1–3 hours on a shaker at room temperature (RT) and destained in lactophenol:ethanol (1∶2) solution at RT overnight. Additionally, leaves were destained in chloral hydrate solution (25 g of chloral hydrate in 10 ml of H_2_O) overnight at RT. Leaves were stored in 70% glycerol, to be examined later and imaged under a Leica DMRB microscope. Whole leaf images were obtained using an Epson scanner.

To inhibit ethylene production, leaves were first infiltrated with 10 µM aminooxiacetic acid (AOA) and incubated for 1 h, and then infiltrated with ToxA or ToxB. Leaves were collected at indicated hours and scanned. Experiments were repeated three times with similar results. Percent damage was estimated as the ratio of the affected area (necrotic or chlorotic) (A_a_) to the total infiltration zone (A_t_) (A_a/_A_t_). The A_a_ was determined by subtracting the unaffected area (A_u_) from A_t_ (A_t_-A_u_) using Adobe Photoshop software as described in [91] with modifications. To define an A_u_ for each experiment we used the Color Range function (under Select) to select the color range of unaffected areas of several leaves with eyedropper tool plus. The “Fuzziness” was adjusted to ensure inclusion of only unaffected tissue in all of the leaves in a single experiment. These settings were saved as the “unaffected area color range settings”. For each leaf, A_t_ was determined by selecting the entire treatment zone with the magic wand tool and recording the total # of pixels from that area (displayed in the Histogram window). Subsequently, the “unaffected area color range settings” were loaded into the Color Range window, the unaffected area selected, and A_u_ was obtained by recording the # of pixels (displayed in the Histogram window).

### Extraction and Quantification of Phenolic Compounds

The accumulation of cytosolic (C) and cell wall-bound (CWB) phenolic compounds for Katepwa (ToxA and ToxB sensitive) and Auburn (ToxA and ToxB insensitive) leaves treated with 2 µM hetToxA, 20 µM hetToxB, or H_2_O at 24 and 48 hpi was determined as described previously [92] with modifications. Briefly, eight leaves were infiltrated per treatment, and 4-cm sections of each infiltration zone were collected, pooled, weighed and stored at −80°C. Later, leaves were ground in liquid nitrogen and dissolved in 500 µL of 50% methanol and incubated for 90 min at 80°C, followed by a 5 min centrifugation at 3000×*g*. The supernatant was used to quantify the C phenolic compounds via the Folin-Ciocalteau assay. The remaining pellets were saved to extract CWB phenolics. The pellets were saponified with 150 µL of 0.5 M NaOH for 24 h, at RT under dark conditions. After the 24 h incubation period was completed, the mixtures were neutralized with 50 µL of 2N HCl and centrifuged for 5 min at 3000×*g*. The supernatants were separated to carry out the Folin-Ciocalteau assay using 25 µl of the sample. The Folin-Ciocalteau assay was performed by adding 50 µL of 2N Folin-Ciocalteau reagent. After 3 min incubation, 100 µL of 20% Na_2_CO_3_ was added to the sample and incubated for 20 min in the dark. The absorbance of the samples was measured at 725 nm. The phenolic content was determined from a standard curve prepared with *p*-coumaric acid (0, 0.01, 0.025, 0.05, 0.1, 0.15, 0.25, 0.5 mg/ml).

### ROS Detection via Nitroblue Tetrazolium (NBT) Staining

NBT staining of 3-cm sections of ToxB- or H_2_O-treated leaves was performed as described previously [93], with the following modifications: the leaves were vacuum infiltrated with NBT at 1 mg/ml in staining buffer (10 mM KPO_4_, 10 mM NaN_3_) for 30 min and further stained overnight at RT. Leaves then were destained overnight in 95% ethanol and visualized on a Leica DMRB microscope.

### Isolation of Thylakoids, Blue Native (BN)-gel and Two-dimensional Gel Electrophoresis

Leaves were infiltrated with either ToxB or H_2_O and collected at 24 and 48 hpi. Thylakoids were isolated and BN-gel and two-dimensional gel electrophoresis was conducted as described [26]. Data presented is representative of three biological replicates.

### Data Availability

All microarray data is deposited at EBI ArrayExpress under accession E-MTAB-963. These data are also available online at http://wheat.cgrb.oregonstate.edu. Software tools used in this study are available at http://mocklerlab-tools.cgrb.oregonstate.edu/.

## Supporting Information

Figure S1 **Venn diagrams showing the comparison among differentially regulated genes identified using four statistical methods.** Venn diagrams show the comparison among differentially expressed genes identified using four methods: Limma [94], [95], SAM [96], PaGE [97], and BRAT (http://brat.cgrb.oregonstate.edu/). Differentially expressed probesets were identified using the following thresholds within each program: LIMMA - corrected p-value <0.01; SAM – false discovery rate (FDR) <2%; PaGE - FDR <12%; BRAT - FDR <10%. **A.** Number of probesets up-regulated at 9, 14, 24, 48 hpi (hours post infiltration). **B.** Number of probesets down-regulated at 9, 14, 24, 48 hpi.
(PPT)

Figure S2 **ToxB microarray validation results with selected probesets.** Expression patterns were validated by RT-PCR (left panel). Graphs represent expression patterns of corresponding probesets for control (open circle) and ToxB-treated (black circle) leaves (right panel). Data represent mean and standard deviation of probeset signal intensities from three biological replicates.
(EPS)

Table S1 **Updated Gene Chip**® **Wheat Genome Array annotation.**
(XLS)

Table S2 **Annotations, regulation, and mean intensitites with standard deviations of statistically significant differentially regulated probesets for ToxB microarray experiment.**
(XLS)

Table S3 **Statistically significant over-represented GO terms for ToxB microarray experiment.**
(XLS)

Table S4 **Comprehensive data of statistically significant differentially regulated probesets of ToxB and ToxA microarray experiments (** **Figures 3** **,** **4** **,** **5** **,** **6** **,** **7** **,** **8** **,** **9** **,** **10** **and related text).**
(XLS)

Table S5 **Statistically significant differentially expressed probsets and their regulation for a selected group of genes for ToxB and ToxA microarray experiments presented in the text.**
(XLS)
