# Supplementary material for: Host-Selective Toxins of Pyrenophora tritici-repentis Induce Common Responses Associated with Host Susceptibility
Source: PLoS One. 2012 Jul 6;7(7):e40240. doi: 10.1371/journal.pone.0040240 (PMC3391247; doi:10.1371/journal.pone.0040240)
Supplement: Figure S1 — Venn diagrams showing the comparison among differentially regulated genes identified using four statistical methods. Venn diagrams show the comparison among differentially expressed genes identified using four methods: Limma [94], [95], SAM [96], PaGE [97], and BRAT (http://brat.cgrb.oregonstate.edu/). Differentially expressed probesets were identified using the following thresholds within each program: LIMMA - corrected p-value <0.01; SAM – false discovery rate (FDR) <2%; PaGE - FDR <12%; BRAT - FDR <10%. A. Number of probesets up-regulated at 9, 14, 24, 48 hpi (hours post infiltration). B. Number of probesets down-regulated at 9, 14, 24, 48 hpi. (PPT) [file pone.0040240.s001.ppt]

## Slide 1
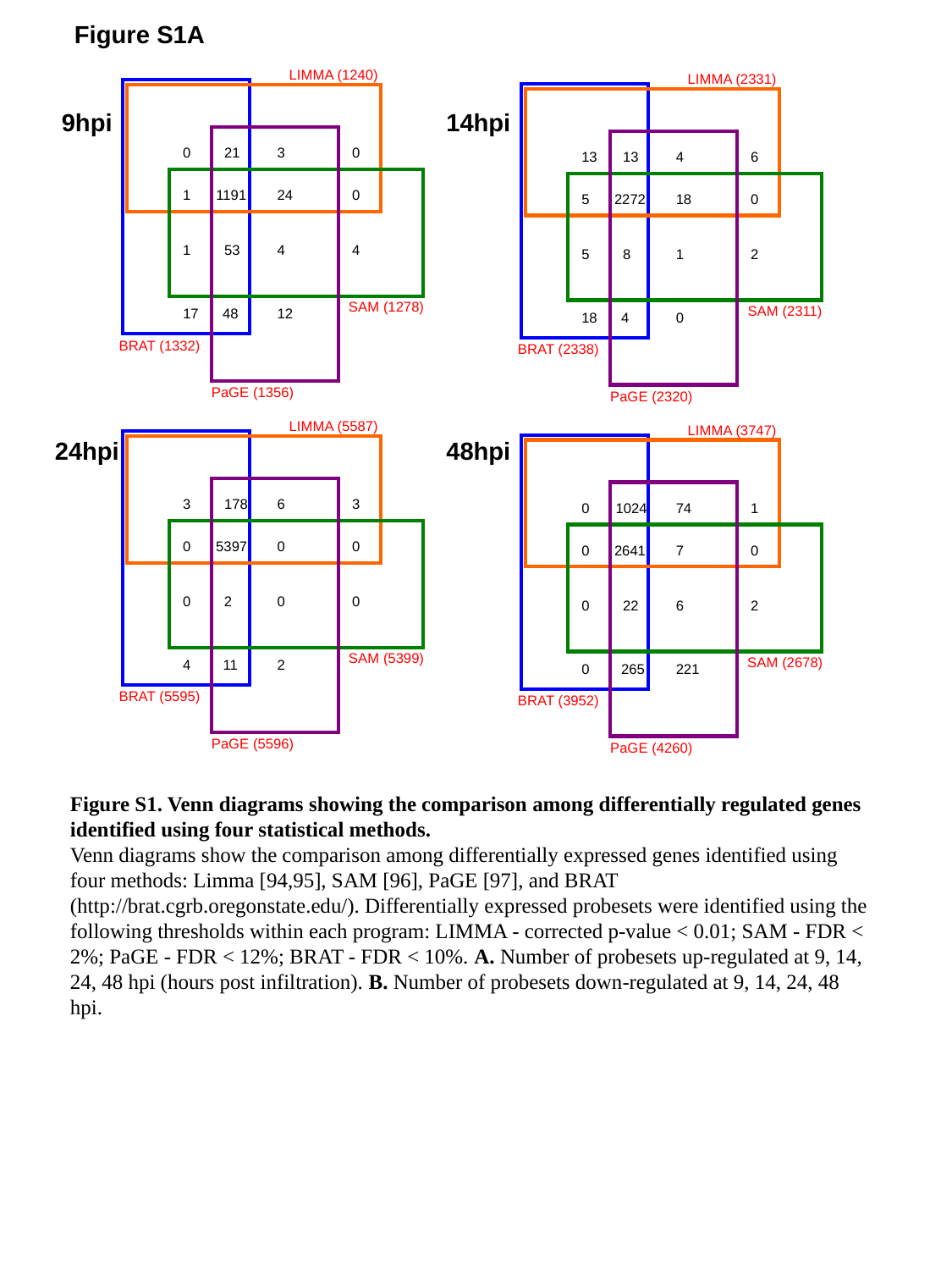

Figure S1A
LIMMA (1240)
LIMMA (2331)
9hpi
14hpi
0
21
3
0
13
13
4
6
1
1191
24
0
5
2272
18
0
1
53
4
4
5
8
1
2
SAM (1278)
SAM (2311)
17
48
12
18
4
0
BRAT (1332)
BRAT (2338)
PaGE (1356)
PaGE (2320)
LIMMA (5587)
LIMMA (3747)
24hpi
48hpi
3
178
6
3
0
1024
74
1
0
5397
0
0
0
2641
7
0
0
2
0
0
0
22
6
2
SAM (5399)
SAM (2678)
4
11
2
0
265
221
BRAT (5595)
BRAT (3952)
PaGE (5596)
PaGE (4260)
Figure S1. Venn diagrams showing the comparison among differentially regulated genes identified using four statistical methods.
Venn diagrams show the comparison among differentially expressed genes identified using four methods: Limma [94,95], SAM [96], PaGE [97], and BRAT (http://brat.cgrb.oregonstate.edu/). Differentially expressed probesets were identified using the following thresholds within each program: LIMMA - corrected p-value < 0.01; SAM - FDR < 2%; PaGE - FDR < 12%; BRAT - FDR < 10%. A. Number of probesets up-regulated at 9, 14, 24, 48 hpi (hours post infiltration). B. Number of probesets down-regulated at 9, 14, 24, 48 hpi.

## Slide 2
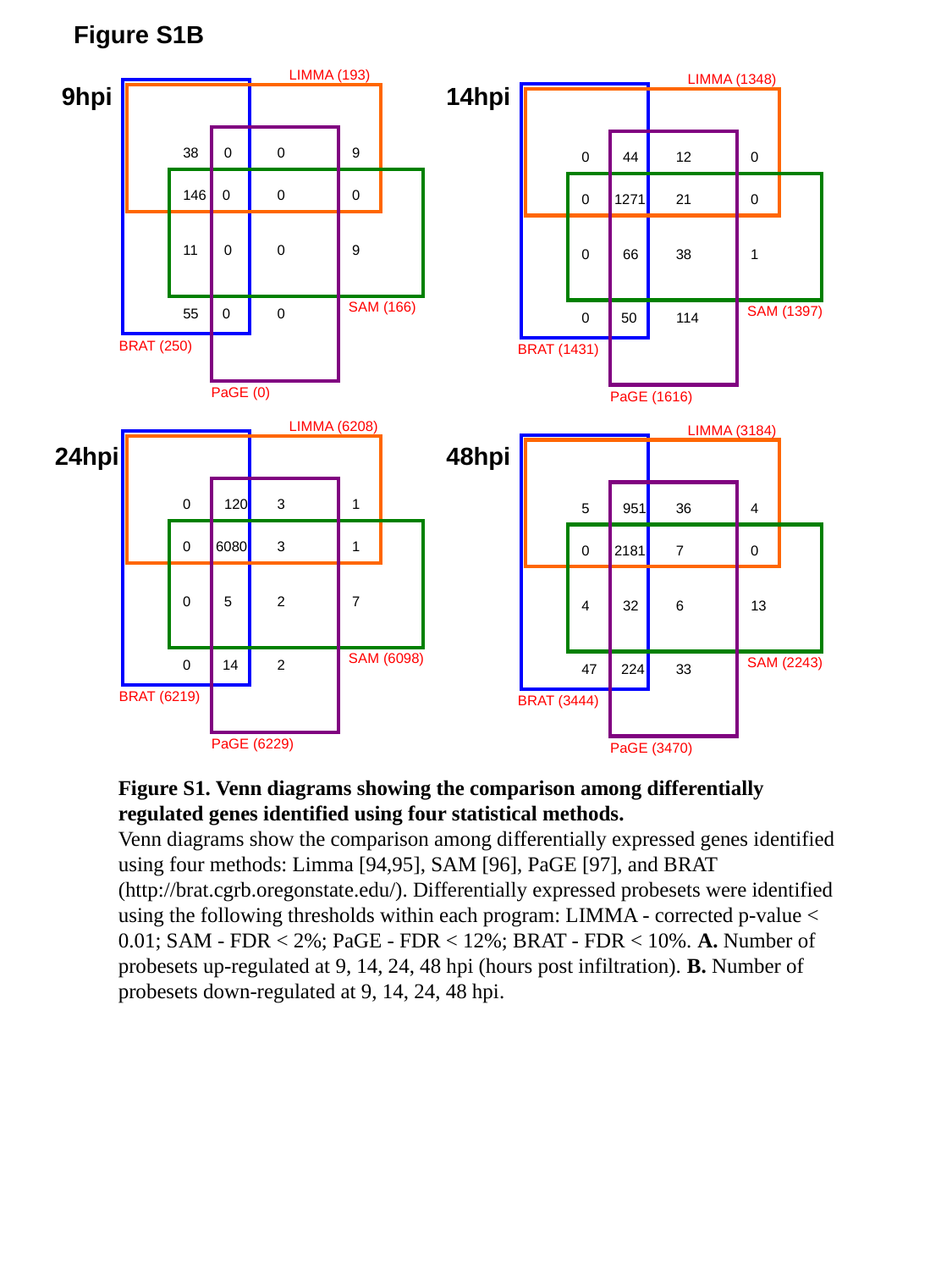

Figure S1B
LIMMA (193)
LIMMA (1348)
9hpi
14hpi
38
0
0
9
0
44
12
0
146
0
0
0
0
1271
21
0
11
0
0
9
0
66
38
1
SAM (166)
SAM (1397)
55
0
0
0
50
114
BRAT (250)
BRAT (1431)
PaGE (0)
PaGE (1616)
LIMMA (6208)
LIMMA (3184)
24hpi
48hpi
0
120
3
1
5
951
36
4
0
6080
3
1
0
2181
7
0
0
5
2
7
4
32
6
13
SAM (6098)
SAM (2243)
0
14
2
47
224
33
BRAT (6219)
BRAT (3444)
PaGE (6229)
PaGE (3470)
Figure S1. Venn diagrams showing the comparison among differentially regulated genes identified using four statistical methods.
Venn diagrams show the comparison among differentially expressed genes identified using four methods: Limma [94,95], SAM [96], PaGE [97], and BRAT (http://brat.cgrb.oregonstate.edu/). Differentially expressed probesets were identified using the following thresholds within each program: LIMMA - corrected p-value < 0.01; SAM - FDR < 2%; PaGE - FDR < 12%; BRAT - FDR < 10%. A. Number of probesets up-regulated at 9, 14, 24, 48 hpi (hours post infiltration). B. Number of probesets down-regulated at 9, 14, 24, 48 hpi.
